# Epigenetic DNA Methylation Signatures Associated With the Severity of Paget’s Disease of Bone

**DOI:** 10.3389/fcell.2022.903612

**Published:** 2022-06-13

**Authors:** Ilhame Diboun, Sachin Wani, Stuart H. Ralston, Omar M. E. Albagha

**Affiliations:** ^1^ Division of Genomic and Translational Biomedicine, College of Health and Life Sciences (CHLS), Hamad Bin Khalifa University (HBKU), Qatar Foundation (QF), Doha, Qatar; ^2^ Translational Genetics and Bioinformatics Section, Research Division, Sidra Medicine, Doha, Qatar; ^3^ Centre for Genomic and Experimental Medicine, Institute of Genetics and Cancer, University of Edinburgh, Edinburgh, United Kingdom

**Keywords:** paget’s disease of bone, bone, epigenetics, DNA methylation, genetics

## Abstract

**Background:** Paget’s disease of bone (PDB) is characterized by focal areas of dysregulated bone turnover resulting in increased bone loss and abnormal bone formation with variable severity. PDB has a complex etiology and both genetics and environmental factors have been implicated. A recent study has identified many differentially methylated loci in PDB compared to healthy subjects. However, associations between DNA methylation profiles and disease severity of PDB have not been investigated.

**Objectives:** To investigate the association between DNA methylation signals and PDB severity.

**Methods:** Using 232 well-characterized PDB subjects from the PRISM trial, a disease severity score was devised based on the clinical features of PDB. DNA methylation profiling was performed using Illumina Infinium HumanMethylation 450K array.

**Results:** We identified 100 CpG methylation sites significantly associated with PDB severity at FDR <0.05. Additionally, methylation profiles in 11 regions showed Bonferroni-significant association with disease severity including six islands (located in *VCL*, *TBX5*, *CASZ1*, *ULBP2*, *NUDT15* and *SQSTM1*), two gene bodies (*CXCR6* and *DENND1A*), and 3 promoter regions (*RPL27*, *LINC00301* and *VPS29*). Moreover, FDR-significant effects from region analysis implicated genes with genetic variants previously associated with PDB severity, including *RIN3* and *CSF1*. A multivariate predictor model featuring the top severity-associated CpG sites revealed a significant correlation (R = 0.71, *p* = 6.9 × 10^−16^) between observed and predicted PDB severity scores. On dichotomizing the severity scores into low and high severity, the model featured an area under curve (AUC) of 0.80, a sensitivity of 0.74 and a specificity of 0.68.

**Conclusion:** We identified several CpG methylation markers that are associated with PDB severity in this pioneering study while also highlighting the novel molecular pathways associated with disease progression. Further work is warranted to affirm the suitability of our model to predict the severity of PDB in newly diagnosed patients or patients with family history of PDB.

## 1 Introduction

Paget’s disease of bone (PDB) is the second most common form of bone disorders in the UK amongst individuals over the age of 50 years (Ralston, 2008). Patients with PDB can develop pathological fractures and deformities in the affected bones with negative effects on quality of life. Other complications include hearing loss, bone pain and rarely osteosarcoma. At the molecular level, PDB is thought to result from abnormal increase in osteoclastic bone resorption activity together with disorganized bone formation by osteoblasts. The bone remodelling process in affected areas no longer serves its natural physiological purpose of renewing old bone and maintaining skeletal strength. Current evidence indicates that the pathology of PDB is complex, involving both genetic and environmental factors. At the genetic level, mutations in the Sequestosome one gene (*SQSTM1*) have been reported in 40–50% of patients with family history of PDB (Ralston and Albagha, 2014). Additionally, Genome-Wide Association Studies (GWAS) have identified several PDB risk loci (Albagha et al., 2010; Albagha et al., 2011), including variants in Optineurin (*OPTN*) (Obaid et al., 2015; Wong et al., 2021), Ras and Rab Interactor 3 (*RIN3*) (Vallet et al., 2015; Vallet et al., 2021) and Promyelocytic leukemia gene (*PML*) (Wani et al., 2022) among others. Environmental factors that have been suggested to be associated with PDB include viral infections from the Paramyxoviridae family (Ralston, 2013), low dietary calcium intake (Siris, 1994) or Vitamin D deficiency (Barker and Gardner, 1974), mechanical loading (Gasper, 1979), exposure to environmental toxins (Adachi et al., 1998; Singer, 2015), and exposure to smoke (Audet et al., 2017). Because of the complex etiology of PDB, early prediction of disease onset remains largely unreliable, calling for further research to uncover efficient predictive biomarkers. From a clinical point of view, it is possible that early intervention may hold the key to preventing disease complications/progression (Cronin et al., 2020).

Numerous studies have explored potential predictors of PDB severity. Importantly, mutations in *SQSTM1* have been associated with increased severity of PDB (Visconti et al., 2010). A previous study by our group derived a polygenic risk allele score, based on alleles previously associated with PDB through GWAS (Albagha et al., 2013). We showed a significant association between the risk allele score and a composite disease severity score in metanalysis of five separate cohorts of PDB patients; with an effect size approximately one third of that observed in patients with mutations of *SQSTM1* (Albagha et al., 2013). The severity score used was devised based on various clinical features of PDB including prior history of bone fractures, orthopedic surgeries for PDB, history of osteosarcoma, bone deformities, use of hearing aids if the patient had PDB of the skull bones, previous bisphosphonate treatments and age at diagnosis. In addition, another study based on 463 PDB patients and 220 controls reported that the circulating concentrations of antibodies directed against a number of Paramyxoviridae viruses including measles virus (MV), respiratory syncytial virus (RSV), canine distemper virus (CDV), mumps, rubella and varicella zoster virus (VZV) were not associated with the presence or severity of PDB (Visconti et al., 2017).

DNA methylation is an important mechanism of epigenetic regulation of gene expression. It involves conversion of cytosine bases in DNA, found adjacent to guanine nucleotide to form CpG sites, into 5-methylcytosine (5mC) by DNA methyltransferases (DNMTs). Methylation regulates gene expression by blocking the binding of transcription factors to promotors or via recruitment of proteins involved in gene repression (Moore et al., 2013). The patterns of DNA methylation are either conserved by maintenance DNMTs (Dnmt1) during DNA replication or can spawn via *de novo* DNMTs (Dnmt3a and Dnmt3b). Importantly, aberrant DNA methylation is associated with various pathologic conditions such as cancers and imprinting disorders (Robertson, 2005), and can also serve as a biomarker for disease progression or may be targeted by epigenetic modifiers for therapeutic benefits (Piekarz and Bates, 2009).

Epigenetic mechanisms play pivotal roles in regulating gene expression, modulating host transcriptomic machinery by invading viruses as well as mediating the influence of environmental factors. Given the evidence that PDB has both environmental and genetic components, investigating DNA methylation patterns in PDB patients is of great interest. In a previous study, the epigenetic profiles from PDB patients were compared to matched healthy controls, which led to the identification of a number of differentially methylated loci (Diboun et al., 2021). The identified loci highlighted several key mechanisms including pathways of bone remodeling, mechanical loading as well as immune response to viral infection. Moreover, a trained multivariate classifier was able to discriminate PDB patients from healthy controls in a cross-validation cohort with high efficiency (Diboun et al., 2021).

In this study, we aimed to identify associations between DNA methylation levels and the extent of disease severity in patients with PDB. Importantly, we identified a multivariate predictor of severity of PDB with high discriminatory performance, which was based on the identification of a panel of differentially methylated loci in PDB patients. Overall, our findings support the potential exploration of epigenetic modifiers for clinical benefits in PDB patients. However, the clinical translation of our findings warrants further investigations.

## 2 Materials and Methods

### 2.1 Study Subjects

This study was based on PDB patients recruited as part of the Paget’s disease Randomised Trial of Intensive versus Symptomatic Management (PRISM) study (ISRCTN12989577) (Tan et al., 2017). The PRISM study is a UK based multicenter study designed to compare the effects of two treatment strategies for PDB; intensive versus symptomatic. Patients with clinical evidence of PDB were assessed at baseline and relevant clinical details were collected which included: the number of bones affected as determined by radionuclide bone scan, history of orthopaedic surgery for PDB, fractures through affected bone, use of a hearing aid in patients with skull involvement, the presence and severity of bone deformity, the age at onset, and details of previous bisphosphonate therapy. Blood samples were taken at the baseline visit before randomisation to the two treatment strategies. Subjects who consented to provide blood sample for DNA analysis and with complete clinical details allowing assessment of PDB severity were included in this study (n = 253). The study was approved by the UK Multicenter Research Ethics Committee for Scotland (MREC01/0/53) and NHS Lothian, Edinburgh (08/S1104/8) ethics review committees. All participants provided written informed consent. The severity of PDB disease was defined as a composite score as previously described (Albagha et al., 2013). Due to the semi-quantitative nature of the phenotype of interest in this study, R package *pwr,* which provides power analysis functions suitable for regression analyses was used. Assuming an effect size of 0.10 (equivalent to the root square of the regression’s anticipated adjusted R^2^), a minimum of 132 subjects was needed to achieve 80% power at array-wide significance levels. Hence, our study was adequately powered to detect methylation correlates of disease severity.

### 2.2 DNA Methylation Profiling

Genomic DNA was extracted from peripheral blood collected in 10 ml EDTA Vacutainers (Becton Dickinson, UK) using QIAamp DNA blood maxi kit (Qiagen, Germany) by following the manufacturer’s protocol. Genomic DNA quality was assessed by NanoDrop spectrophotometer (Thermo Scientific, UK) and quantified using PicoGreen dsDNA assay (Invitrogen, UK). Genome-wide DNA methylation measurements were determined using Illumina Infinium HumanMethylation 450K array (Illumina, United States) by following the manufacturer’s protocol. Briefly, the Zymo EZ-96 DNA methylation Kit (Zymo Research, United States) was used to perform bisulphite conversion on 500 ng of DNA. Bisulphite converted DNA was then amplified, fragmented, and hybridized to Illumina Infinium HumanMethylation 450K Beadchip using standard Illumina protocol.

### 2.3 Methylation Data Analysis

#### 2.3.1 Pre-processing

Raw methylation data were analyzed using the R RnBeads package (Müller et al., 2019). Details of data pre-processing steps have been described previously (Diboun et al., 2021). Briefly, Samples with low methylated or unmethylated median intensity (<11.0) were excluded (*n* = 35) and CpG sites that were cross reactive, containing single nucleotide polymorphisms (SNPs), located on sex chromosome or those associated with epigenetic effects of smoking were removed, leaving 232 samples with 429,156 CpG sites for downstream analyses. Estimation of cell type composition was performed using the Houseman reference method (Houseman et al., 2012) and included CD14 monocytes, CD19 B-cells, CD4 T-cells, CD56 NK cells, CD8 T-cells, eosinophils, granulocytes, and neutrophils. Surrogate variables analysis (SVA) was used to estimate the unmeasured confounder effects by quantifying multivariate signatures that do not explain the phenotype of interest. Background correction and across-array normalization were achieved using the Enmix (Pidsley et al., 2013) and the SWAN methods respectively, implemented as part of the RnBeads package. For all downstream analysis, the M values, calculated as log_2_ ((methylated signal+1)/(unmethylated signal+1)), were used. All chromosomal positions are presented in reference to Genome Reference Consortium Human Build 37 (GRCh37).

#### 2.3.2 Statistical Analysis of Methylation Data

Two different linear models were used to handle the methylation levels from individual CpG sites as well as the global methylation profiles from genomic regions, where a region refers to either CpG island, promoter or gene body. The delineation of the CpG islands was based on the Illumina array manifest file. Sites not annotated to an island by the illumina manifest were assigned to “promoter” regions if they mapped to transcription start site (TSS) and those within gene sequences were assigned to “gene body” region. With the individual CpG sites, we used the General Linear Model with Bayesian variance optimization provided by the R Limma package (Smyth, 2004), to regress the site methylation level on PDB_severity and confounders according to the following model:

*site ∼ confounder + PDB_severity*

Where the term *confounder* refers to the collective effect of age, sex, array, bisulfide conversion batch, array scan batch, blood cell type composition and the top ten SVA components.

For a given region with *n* CpG sites, a Generalized Linear Model was applied in two steps: First, the Poisson-distributed PDB severity score was regressed on confounders alone. Next, it was regressed on confounders in addition to all *n* sites within the region. The difference in the deviance (equivalent of residuals in the linear model) between the two models was a Chi-square distributed variable with *n* degrees of freedom and a *p*-value for this variable was derived accordingly. Effectively, the *p*-value indicates the improvement in the fit brought about by the inclusion of the sites in the model, whilst normalizing for their number. The two-step Generalized Linear Model has been described in (Diboun et al., 2021) and is here summarized as:

(step1) *Severity* ∼ *confounder*
(step2) *Severity* ∼ *confounder + site*_*1*_ *+site*_*2*_ *… ….+site*_*n*_

Visualization of selected significant regions within their genomic context was performed using the coMET R package (Martin et al., 2015). Differences in PDB severity scores between males and females were analyzed using unpaired Student’s t-test. One way analysis of variance was used to test differences in severity scores among subjects in different age groups.

#### 2.3.3 Validation of CpG Sites

We assessed if the methylation profiles for CpG sites identified from this study showed any correlations between blood and bone tissue, using previously published data of 28,549 highly correlated CpG sites (*r* ^2^ > 0.74, FDR <0.05) reported by Ebrahimi *et. al.,* (Ebrahimi et al., 2021). We also checked the list of identified sites against the BIOS QTL database (Bonder et al., 2017) to identify any evidence of associations with the expression of nearby genes. Annotation of chromatin state for genomic segments was inferred using ChromHMM using ChIP-seq data from the lymphoblastoid cell line GM12878 (Ernst and Kellis, 2017). Finally, we mapped the list of sites under scrutiny to the nearest genes using the Illumina manifest file and further derived the associated gene ontology (GO) biological processes and ingenuity pathway analysis (IPA) categories. With each set of categories, the number of significant genes mapping to a specific category was compared to the expected category considering its overall frequency on the array, based on the Fisher’s exact test statistics. We used the same approach to perform enrichment analysis of PDB-associated genomic regions from previously published linkage analysis, including 2q36 (chr2:221500001–231000000) (Hocking et al., 2001), 5q31 (chr5:130600001–144500000) (Laurin et al., 2001), 5q35 (chr5: 168500001–180915260) (Hocking et al., 2001; Laurin et al., 2001), and 10p13 (chr10: 12200001–17300000) (Hocking et al., 2001) and the 500 kb regions surrounding GWAS significant SNPs (Albagha et al., 2011): rs10494112 (chr1:110102477–110602477), rs4294134 (chr7:135043128–135543128), rs2458413 (chr8:105109432–105609432), rs1561570 (chr10:12905726–13405726), rs10498635 (chr14:92853309–93353309), rs5742915 (chr15:74086633–74586633), and rs3018362 (chr18:59832033–60332033).

### 2.4 Multivariate Predictors of PDB Severity

The Orthogonal Partial Least Square (OPLS) algorithm by SIMCA ver.15 (Umetrics, Sweden) was used to construct predictive models. For this analysis, the study cohort was randomly divided into two sets; *discovery set* (*n* = 116) and *cross-validation set* (*n* = 116)*.* The sites included in the model were derived from pooling the significant sites from site and region-level analyses restricted to the discovery set to avoid any bias, which led to a pooled list of 2,247 sites from the discovery set. The model was then tested on the cross-validation set. Additionally, we ran the Lasso and Elastic-Net regularized Generalized Linear Model, implemented in R package Glmnet, to delineate a subset of sites that still corresponds to a significant proportion of variation in PDB severity in the discovery set (Friedman et al., 2010). The models were then tested on the cross-validation set*.* Additionally, considering the continuous nature of our phenotype, correlation analysis was performed to compare the predicted versus observed PDB severity scores. The study subjects were also divided into two groups; low_severity (severity score <7) or high_severity (severity score ≥7) based on the median value of 7. Receiver operator characteristics (ROC) curve analysis was performed, and summary statistics were calculated to determine the area under curve (AUC), specificity and sensitivity values.

## 3 Results

### 3.1 Characteristics of Study Subjects

The characteristic features and descriptive statistics of study subjects are presented in Table 1. The average (± standard deviation) age at the time of study was 72.3 (±8.1) years and age at diagnosis was 62.1 (±10.9) years. The number of male subjects was slightly higher than female, but this was not statistically significant (*p* = 0.55). The PDB severity score ranged from 2 to 18 with a mean of 6.6. We found no significant difference in PDB severity scores between male and female subjects (*p* = 0.13) or between the age groups listed in Table 1 (*p* = 0.49).

**TABLE 1:** Characteristic features of study subjects.

Characteristic	PDB* patients
Number	232
Gender (Male:Female)	124:108
Age (years)	72.3 ± 8.1
Age at Diagnosis (years)	62.1 ± 10.9
PDB severity Score (n)**	
All subjects	6.6 ± 2.8 (232)
Males	6.9 ± 3.1 (124)
Females	6.3 ± 2.6 (108)
Age 30–39 years	8.0 (1)
Age 40–49 years	8.3 ± 2.1 (3)
Age 50–59 years	7.4 ± 2.0 (13)
Age 60–69 years	6.7 ± 2.3 (55)
Age 70–79 years	6.4 ± 3.0 (116)
Age 80–89 years	6.7 ± 3.3 (44)
*SQSTM1* Mutation	33

* Paget’s disease of bone. Data are presented as Mean ± SD.

** PDB, severity scores were not signficantly different between male and female subjects (p = 0.13) or between the listed age groups (p = 0.49).

### 3.2 DNA Methylation Sites Associated With Disease Severity

**FIGURE 1:**
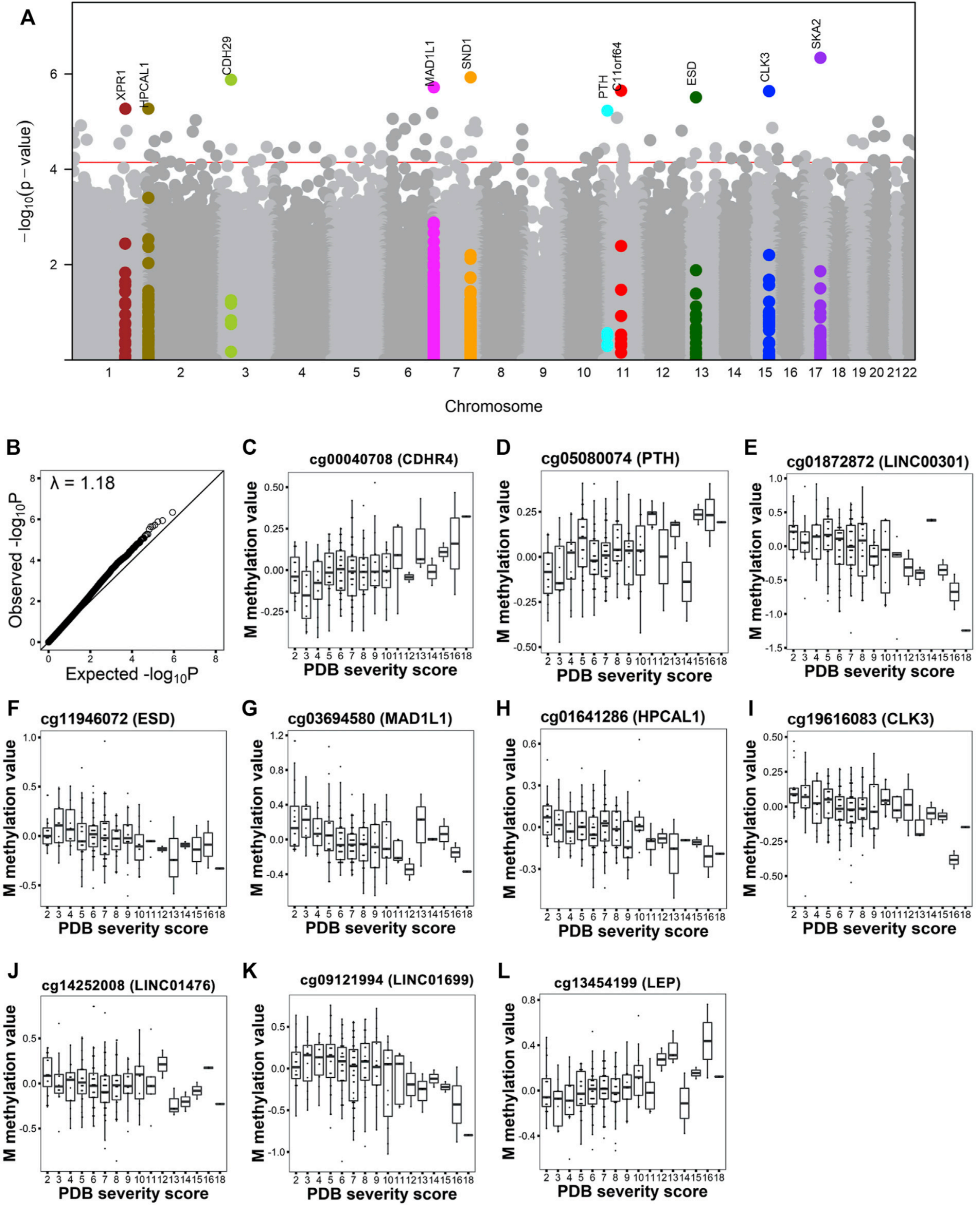
Site-level association analysis with PDB severity. **(A).** Manhattan plot shows the CpG sites associated with PDB severity at FDR <0.05 (red horizontal line). The top 10 FDR-significant CpG sites are highlighted in color and annotated with the closest gene. **(B)**. Q-Q plot shows the expected versus observed -log_10_ P. Lambda indicates the genomic inflation factor. **(C–L)** Box and whiskers plots show PDB severity versus methylation levels from the top 10 sites after correction for confounders.

We used multiple linear models to identify associations between PDB severity score and methylation at 429,156 CpG sites. Following multiple testing corrections, 100 sites showed significant associations with disease severity at FDR <0.05 (Figure 1A and Supplementary Table S1). The overall inflation factor was 1.18 (Figure 1B). Table 2 lists the top 10 most significant sites together with CpG sites with lower ranks of the 100 FDR-significant sites that were located within or near genes with known bone functions.

**TABLE 2:** A subset of FDR-significant CpG sites from site level association analysis with PDB severity. Listed are the top 10 most significant sites together with CpG sites with lower ranks that were located genes with known bone functions.

CpG Site	Chr	Position	Beta^†^	*p*-Value	FDR	Gene	Location^‡^	ChromHMM	Rank
cg14252008	17	57598958	-0.040	4.60 × 10^−7^	0.016	LINC01476*	Body	8	1
cg13454199	7	127914981	0.027	1.16 × 10^−6^	0.016	LEP*	Downstream	13	2
cg00040708	3	49838627	0.023	1.31 × 10^−6^	0.016	*CDHR4*	TSS1500	13	3
cg03694580	7	2271722	-0.035	1.91 × 10^−6^	0.016	*MAD1L1*	5′UTR	1	4
cg01872872	11	60382923	-0.050	2.26 × 10^−6^	0.016	*LINC00301*	TSS1500	13	5
cg19616083	15	74908514	-0.022	2.30 × 10^−6^	0.016	*CLK3*	Body	1	6
cg11946072	13	47371514	-0.029	3.07 × 10^−6^	0.019	*ESD*	TSS200	1	7
cg01641286	2	10483916	-0.021	5.33 × 10^−6^	0.025	*HPCAL1*	5′UTR	13	8
cg09121994	1	181205431	-0.044	5.37 × 10^−6^	0.025	LINC01699*	Upstream	13	9
cg05080074	11	13517684	0.022	5.92 × 10^−6^	0.025	*PTH*	TSS200	13	10
cg05094429	6	167536184	0.025	6.55 × 10^−6^	0.025	*CCR6*	5′UTR	4	11
cg01132064	11	46382562	-0.025	8.39 × 10^−6^	0.026	*DGKZ*	Body	4	12
cg19311431	2	162091931	0.034	1.64 × 10^−5^	0.027	*TANK*	Body	11	26
cg03014008	20	57463767	0.027	2.01 × 10^−5^	0.028	*GNAS*	Body	1	30
cg23635663	22	30639979	0.040	2.44 × 10^−5^	0.028	*LIF*	Body	10	37
cg20609803	1	161184305	-0.015	2.85 × 10^−5^	0.029	*FCER1G*	TSS1500	10	42
cg03349241	8	145743334	-0.023	3.11 × 10^−5^	0.029	*RECQL4*	TSS200	6	46
cg03177593	3	171890144	-0.027	3.40 × 10^−5^	0.029	*FNDC3B*	Body	7	49
cg18362496	11	2019930	0.013	3.71 × 10^−5^	0.029	*H19*	TSS1500	8	54
cg24335984	3	50361554	0.018	3.79 × 10^−5^	0.029	*HYAL2*	TSS1500	11	56
cg06217803	7	128830440	0.021	4.23 × 10^−5^	0.030	*SMO*	Body	6	59
cg19327006	6	143084770	0.017	5.20 × 10^−5^	0.040	*HIVEP2*	Body	11	69
cg17359265	5	178567126	-0.017	5.39 × 10^−5^	0.040	*ADAMTS2*	Body	13	71
cg08452338	3	149271990	0.028	6.29 × 10^−5^	0.046	*WWTR1*	Body	11	84
cg07341934	20	57463711	0.022	6.50 × 10^−5^	0.046	*GNAS*	Body	1	89
cg17775490	20	45179354	-0.026	7.18 × 10^−5^	0.049	*OCSTAMP*	TSS200	6	100

* CpG was manually mapped to the closest gene. The rest of the sites were mapped by Illumina ^†^The beta value refers to the effect size from the linear model. Positions are in reference to GRCh37 genome build.^‡^Location relative to gene based on the longest isoform, TSS (Transcription Start Site), UTR (untranslated regions). ChromHMM, indicates chromatin state in the lymphoblastoid cell line GM12878: 1 active promoter, 2 weak promoter, 3 inactive promoter, 4 & 5 strong enhancer, 6 & 7 weak enhancer, 8 insulator, 9 transcriptional transition, 10 transcriptional elongation, 11 weak transcribed, 12 polycomb-repressed, 13 heterochromatin, 14 & 15 repetitive/copy number variation.

Figure 1C–L shows DNA methylation values in relation to disease severity score for the top 10 hits. Amongst these were CpG sites mapping to transcription start site (TSS) regions that showed increasing methylation levels with disease severity. These included cg00040708 (Figure 1C) in cadherin related family member 4 (*CDHR4*) and cg05080074 (Figure 1D) in parathyroid hormone (*PTH*). Conversely, cg01872872 (Figure 1E) at TSS of long intergenic non-protein coding RNA 301 (*LINC00301*) and cg11946072 (Figure 1F) at TSS of Esterase D (*ESD*) showed a decrease in their methylation levels with increasing disease severity. A decrease in methylation level was also observed with increased severity score for two 5′UTR sites; cg03694580 (Figure 1G) in mitotic arrest deficient one Like 1 (*MAD1L1*) and cg01641286 (Figure 1H) in hippocalcin like 1 (*HPCAL1*); in addition to two sites located at gene body; cg19616083 (Figure 1I) in CDC like kinase 3 (*CLK3*) and cg14252008 (Figure 1J) in LINC01476. Additionally, cg09121994 located upstream LINC01699 (Figure 1K) also showed decrease in methylation with increase in severity, while cg13454199 located downstream leptin (*LEP*) (Figure 1L) showed increase in methylation with PDB severity.

**FIGURE 2:**
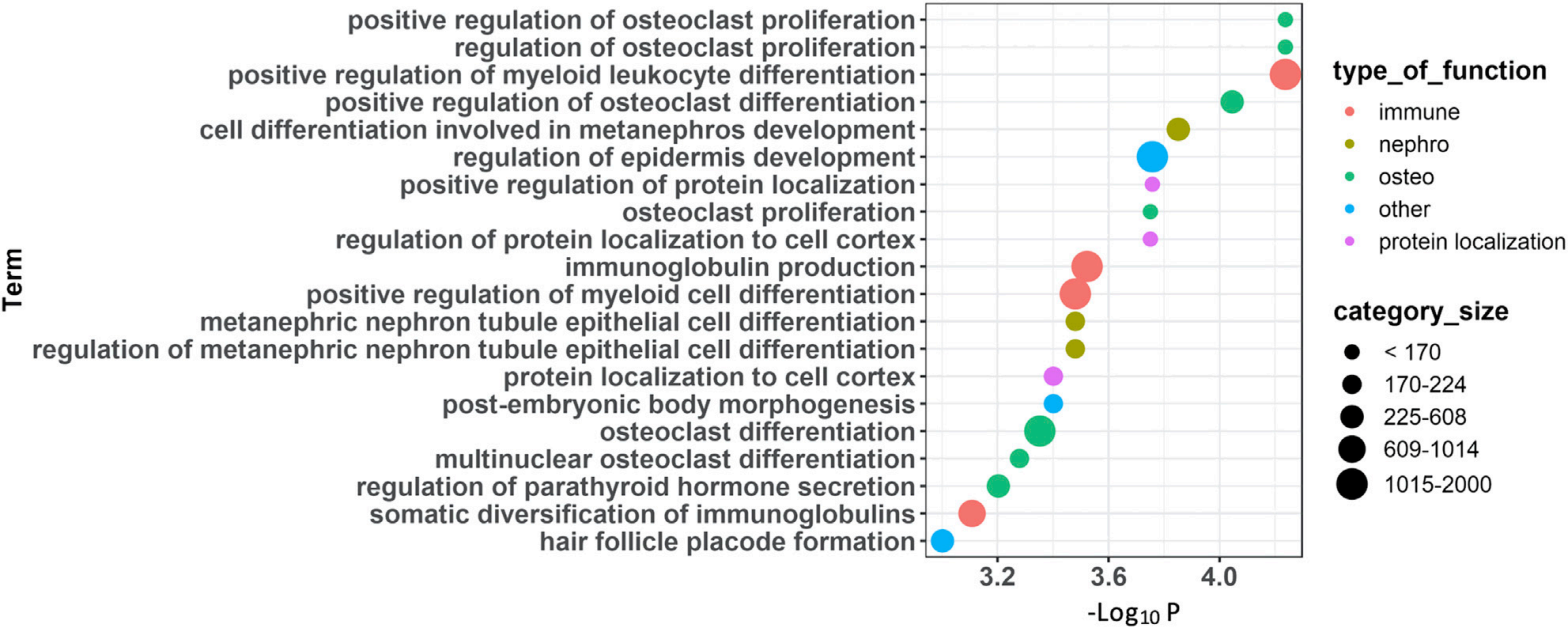
Functional annotations of CpG sites associated with PDB severity. Summary of enrichment analysis of gene ontology (GO) functional categories associated with genes located in the 100 FDR-significant sites.

GO enrichment analyses based on the top 100 FDR-significant sites revealed bone related biological functions including: ‘*positive regulation of osteoclast proliferation’* (GO:0090290, FDR = 0.01) as well as ‘*osteoclast proliferation’* (GO:0002158, FDR = 0.02) and ‘*multinuclear osteoclast differentiation’* (GO:0072674, FDR = 0.04) (Figure 2, Supplementary Table S2). These osteoclast-related functions featured FDR significant CpG sites located within or near genes involved in osteoclast function such as Fc epsilon receptor Ig (*FCER1G*), parathyroid hormone (*PTH*), osteoclast stimulatory transmembrane protein (*OCSTAMP*), TRAF family member-associated NF-kappa B activator (*TANK*), leukemia inhibitory factor (*LIF*), and human immunodeficiency virus type 1 enhancer-binding protein 2 (*HIVEP2*). Other enriched GO biological process terms included immune-related processes (Figure 2, Supplementary Table S2). Enrichment analysis based on IPA further identified ‘*morphology of bones’,* ‘*differentiation of bone cells’* and ‘*Quantity of bone’* associated with FDR significant sites from genes related to bone metabolism such as: disintegrin and metalloproteinase with thrombospondin Motifs 2 (*ADAMTS2*), C-C Chemokine Receptor Type 6 (*CCR6*), the imprinted maternally expressed transcript *H19*, Hyaluronidase 2 (*HYAL2*), kinesin family member 3A (*KIF3A*), and smoothened, frizzled class receptor (*SMO*). In line with the GO enrichment analysis, immune-related functions were also enriched in IPA (Supplementary Table S3)*.*

### 3.3 DNA Methylation Regions Associated With Disease Severity

**FIGURE 3:**
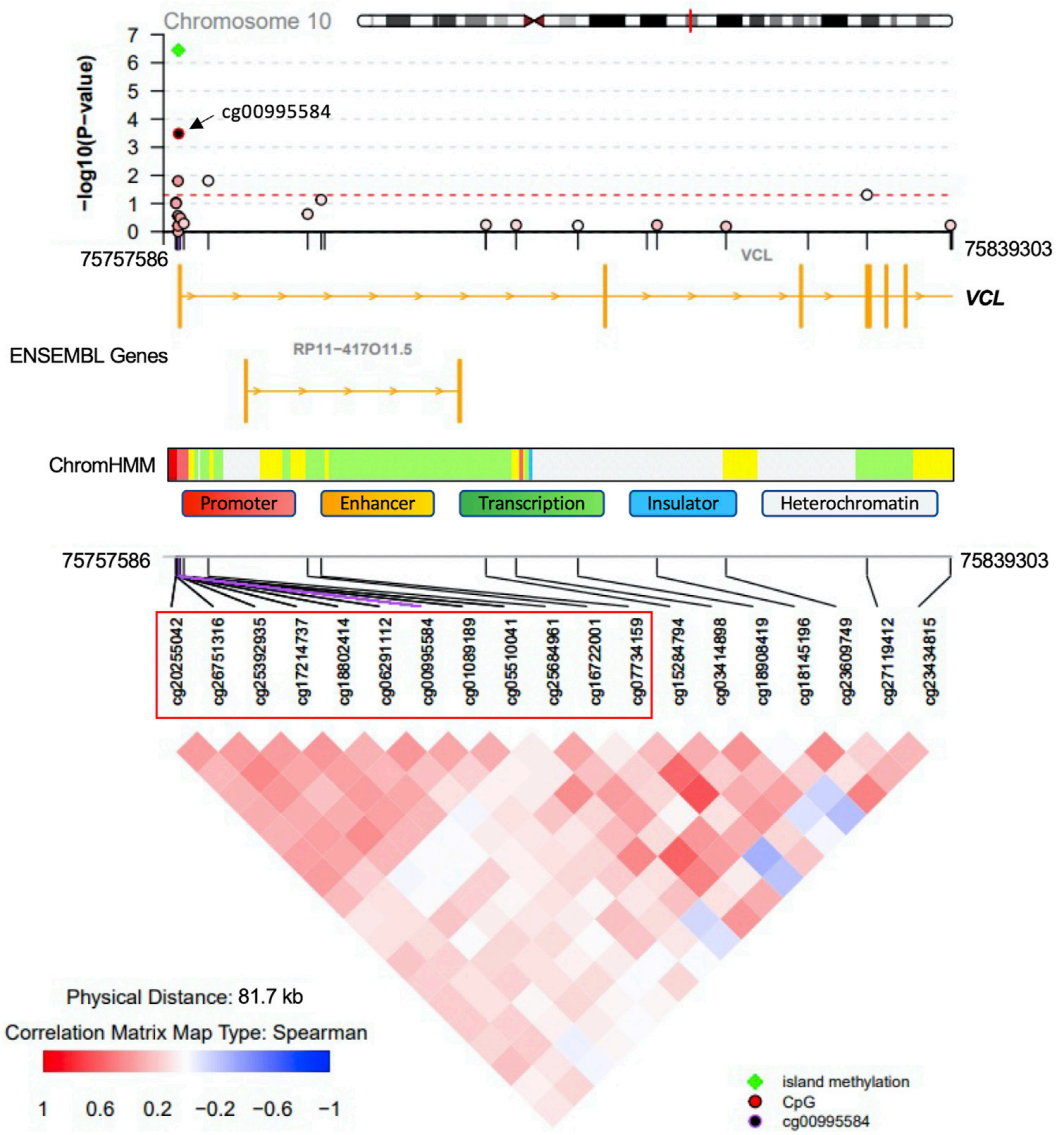
*VCL* Island-level association analysis with PDB severity. The top Bonferroni significant island was located in the promoter region of the *VCL* gene. Green indicates significance from island level analysis The island encompasses 12 CpG sites showing a synergy of methylation effects. Cg00995584 is the most significantly associated site with PDB severity and the colour code in the top regional association plot indicates the correlation levels between each other site and the reference site cg00995584. Ensembl genes are shown in the middle part (orange boxes indicate exons). ChromHMM indicates chromatin state in the lymphoblastoid cell line GM12878. The bottom part denotes the correlation level for all possible pairs of CpG sites.

**FIGURE 4:**
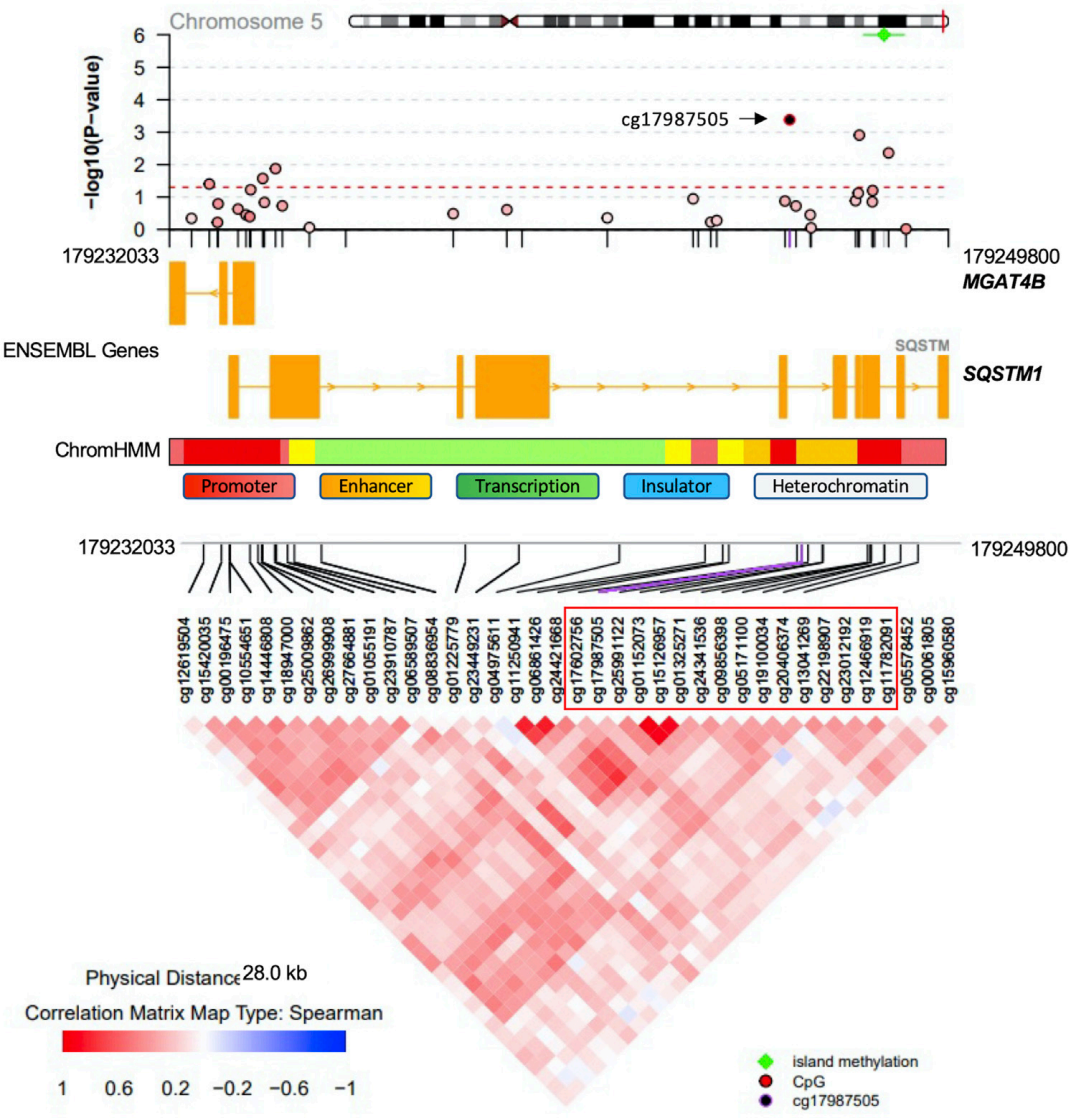
*SQSTM1* Island-level association analysis with PDB severity. The Bonferroni-significant island on chromosome five overlapping with the *SQSTM1* gene. Green indicated significance from island level analysis. The island encompasses 16 CpG sites showing a synergy of methylation effects. Cg17987505 is the most significantly associated site with PDB severity and the color code in the top regional association plot indicates the correlation levels between each other site and the reference site cg17987505. Ensembl genes are shown in the middle part (orange boxes indicate exons). ChromHMM indicates chromatin state in the lymphoblastoid cell line GM12878. The bottom part denotes the correlation level for all possible pairs of CpG sites.

Methylation sites within CpG islands were collectively analyzed using the Poisson family of Generalized Linear Models, which identified six significant islands with a Bonferroni corrected *p*-value < 0.05. These islands were mapped to the following genes: vinculin (*VCL*), T-Box transcription factor 5 (*TBX5*), castor zinc finger 1 (*CASZ1*), UL16 binding protein 2 (*ULBP2*), nudix hydrolase 15 (*NUDT15*) and sequestosome 1 (*SQSTM1*) (Table 3). The regional plots in Figures 3,4 illustrate the synergy in the regulation of methylation levels of adjacent sites within the Bonferroni significant islands of *VCL* and *SQSTM1,* respectively.

**TABLE 3:** Statistically significant islands, gene bodies and promoters from region-level association analysis with PDB severity (all with Bonferroni corrected *p* < 0.05).

Region	Chr	Start	End	# Sites	*p*-Value	FDR	Gene
Island	10	75755183	75761004	12	3.56 × 10^−7^	0.004	*VCL*
Island	12	114839007	114840942	4	5.34 × 10^−7^	0.004	*TBX5*
Island	1	10750680	10754162	6	5.77 × 10^−7^	0.004	*CASZ1*
Island	6	150262044	150266173	12	7.12 × 10^−7^	0.004	*ULBP2*
Island	13	48608110	48616161	16	1.00 × 10^−6^	0.004	*NUDT15*
Island	5	179246001	179251299	16	1.00 × 10^−6^	0.004	*SQSTM1*
Gene body	3	45969823	46000900	17	1.02 × 10^−6^	0.012	*FYCO1/CXCR6*
Gene body	9	126145211	126157693	26	2.11 × 10^−6^	0.012	*DENND1A*
Promoter	17	41149985	41150394	7	1.09 × 10^−6^	0.012	*RPL27*
Promoter	11	60382082	60382923	3	1.32 × 10^−6^	0.012	*LINC00301*
Promoter	12	110939283	110941087	9	1.83 × 10^−6^	0.012	*VPS29*

Positions are in reference to GRCh37 genome build.

Using less stringent multiple testing correction (FDR <0.05) revealed 783 significant island regions (Supplementary Table S4). Amongst non-singleton biological process GO categories tested for enrichment in island regions, top enriched categories included ‘*immune system process’* (GO:0002376; FDR = 0.009), ‘*translation’ (GO:0,006,412; FDR = 0.009), ‘regulation of translation in response to stress’ (GO:0043555; FDR = 0.01)* and ‘*negative regulation of interleukin-10 secretion’ (GO:2001180; FDR = 0.01)*. IPA categories enrichment analysis further highlighted ‘*differentiation of granulocyte progenitors; FDR = 0.001’*, *‘Paget’s disease of bone; FDR = 0.004′* and ‘*abnormal morphology of bone; FDR = 0.002’*.

Further region-based analysis revealed two Bonferroni significant gene body regions; FYVE/coiled-coil domain autophagy adaptor 1 (*FYCO1*) and DENN domain containing 1A (*DENND1A*) (Table 3). Using FDR <0.05 identified 768 genes associated with PDB severity (Supplementary Table S5). Amongst these, the most prominent findings were Ras and Rab interactor 3 (*RIN3*), *SQSTM1* and the colony stimulating factor 1 (*CSF1*); all previously implicated in PDB through GWAS studies (Albagha et al., 2010; Albagha et al., 2011; Albagha et al., 2013). Of note, GO enrichment analysis based on the 768 FDR significant gene bodies did not identify any significantly enriched pathway. However, IPA Enrichment analysis highlighted mostly immune- and viral-related categories including ‘*Degranulation of neutrophils; FDR = 0.004’*, ‘*activation of bone cell lines; FDR = 0.004’,* ‘*Infection by RNA virus; FDR = 0.004’,* and ‘*Ubiquitination of protein; FDR = 0.004’*.

Finally, three promoter regions were associated with PDB severity at Bonferroni corrected *p*-value < 0.05 (Table 3). These were mapped to ribosomal protein L27 (*RPL27*), long intergenic non-protein coding RNA 301 (*LINC00301*) and vascular protein sorting-associated protein 29 (*VPS29*) (Table 3). Using less stringent multiple testing correction (FDR<0.05) revealed 303 significant promoter regions (Supplementary Table S6). These were analyzed for functional enrichment analysis based on GO, which revealed ‘*response to topologically incorrect protein*’ (*GO:0035966; FDR = 0.006*) and ‘*response to unfolded protein’ (GO:0,006,986; FDR = 0.007)* categories; among others. Additionally, IPA enrichment analysis of the same promoter-associated genes highlighted ‘*Endocytosis by monocytes; FDR = 0.003’* and ‘*cell viability of bone cell lines; FDR = 0.007*’, among others.

### 3.4 Validation of Methylation Markers Associated With PDB Severity

To functionally validate our results from association analysis, we searched the BIOS QTL (Bonder et al., 2017) database. We found evidence of methylation-induced cis-regulation of gene expression for three of our FDR-significant sites: cg05094429 was associated with CCR6 expression (*p*-value = 2.5 × 10^−24^, FDR<0.05), cg06320150 was associated with *C22orf34* expression (*p*-value = 2.0 × 10^−4^, FDR = 0.035), while cg00875541 was strongly associated with *C4A* expression (*p*-value = 9.4 × 10^−8^, FDR<0.05). Additionally, nine CpG sites within the Bonferroni-significant gene body region of *CXCR6* were significantly associated with its expression. Amongst these nine sites, cg05705212 was the most significant (*p*-value = 7.9 × 10^−16^, FDR<0.05).

Since the cell types involved in PDB pathogenesis are integral to the bone tissue, we compared our list of CpG sites associated with PDB severity from site and region-level analysis with a published list of highly concordant methylation sites between the blood and bone tissues (refer to methods). We found that 10 out of the 26 FDR-significant genes (listed in Table 2) featured a methylated site that is highly concordant between bone and blood tissue based on the published list (R^2^ > 0.74; FDR <0.05). As for the region-level analysis, five out of the 11 Bonferroni-significant regions listed in Table 3 were associated with blood/bone highly concordant CpG sites (*r* ^2^ > 0.74; FDR <0.05): *TBX5* (3 out of four sites), *CASZ1* (2 out of six sites), *ULBP2* (2 out of 12 sites), *NUDT15* (1 out of 16 sites) and *RPL27* (1 out of 27 sites) (Supplementary Table S7)

Finally, we tested whether our identified methylation markers were frequently mapping to previously identified PDB-linked genomic regions and PDB-associated GWAS regions (refer to methods). We found an overrepresentation of FDR-significant CpG sites and FDR-significant gene bodies in chromosome 5q31 region (*p*-value = 0.034) as well as 5q35 region (*p*-value = 0.05) from the site-level and gene-body level analyses respectively. In contrast, no significant over-representation of the identified methylation markers in previously reported PDB-susceptibility loci identified by GWAS (Albagha et al., 2010; Albagha et al., 2011).

### 3.5 Multivariate Predictive Analysis

**FIGURE 5:**
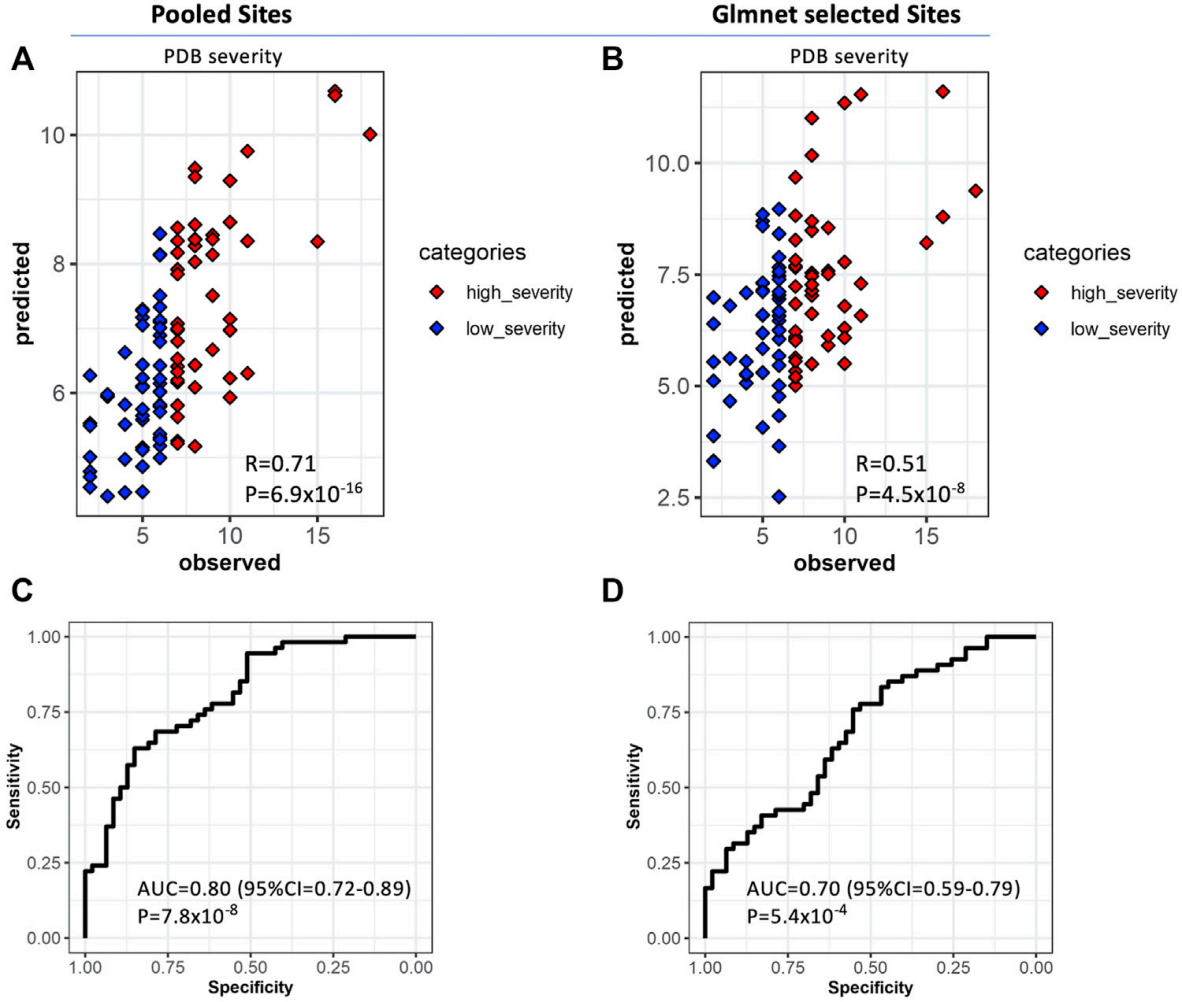
Validation of predictive models for PDB severity scores. Orthogonal Partial Least Square (OPLS) classifier was trained on the discover set and tested on the cross-validation set. The features consisted of the list of significant sites from site and region level analysis based on the discovery samples (Panels A and C, *n* = 2,247 CpG sites) and separately, on a best explanatory subset from this pool of sites obtained with the Glmnet (Panels B and D, n = 33 CpG sites) **(A,B)** Scatter plots of predicted versus observed PDB_severity scores for each model. The observed PDB severity scores were also dichotomized into two groups based on the median PDB severity score of seven; low severity (PDB_severity_score <7; blue) and high severity (PDB_severity_score ≥7; red) and subsequently used for ROC curve analysis showing the area under curve AUC for pooled sites **(C)** and Glmnet selected sites **(D)**.

One of our primary aims was to assess the performance of the methylation sites identified in this study in predicting disease severity scores of PDB. Therefore, we randomly divided our study cohort into discovery (*n* = 116) and cross-validation (*n* = 116) sets. Site and region-level analysis were performed on data from the discovery cohort, which yielded a set of 2,247 significant pooled list of sites. Importantly, the predicted PDB severity scores by the OPLS model trained on this subset of sites were significantly correlated with their observed scores (R = 0.71, *p* = 6.9 × 10^−16^) when tested in the cross-validation set (Figure 5A). We then dichotomized the cohorts into low severity (Score <7) and high severity (Score ≥7) based on the median severity score of 7. When this model was tested on the cross-validation set, the AUC, specificity and sensitivity values were 0.80, 0.74 and 0.68 respectively (Figure 5C). We then prioritized the list of significant pooled list of sites using Glmnet, which resulted in 33 CpG sites. This approach offers a reasonable balance between the number of sites and the predictive capacity of the model. Analysis using the 33 CpG sites resulted in lower correlation compared to the pooled list but remained statistically significant (R = 0.51, *p* = 4.52 × 10^−8^; Figure 5B). Correspondingly, the results from the ROC curve analysis were also less optimal (AUC = 0.7, specificity = 0.64, sensitivity = 0.58; Figure 5D). The list of the 33 Glmnet sites and their annotations are presented in Supplementary Table S8.

## 4 Discussion

We investigated associations between DNA methylation levels and disease severity in PDB patients. Association analysis was conducted at the level of CpG sites and regions. At the level of sites, there were 100 significant associations at FDR <0.05. The top 10 CpG sites were mapped to the following genes: Leptin (*LEP*) which has been shown to regulate bone mass in mice through the central nervous system (Ducy et al., 2000; Karsenty, 2006) and it inhibits human and mouse osteoclast differentiation *in vitro* (Holloway et al., 2002), *CDHR4* a member of the cadherin superfamily which has no known function in bone metabolism, *MAD1L1* whose methylation profile in cancellous bone was shown to correlate with osteoporosis in a cohort of postmenopausal women (Zhou et al., 2020), *CLK3* which is involved in pre-mRNA splicing (Duncan et al., 1998), *ESD* which has a predicted role in glutathione metabolism while a recent study has shown that glutathione promotes osteoclast differentiation (Fujita et al., 2019), *HPCAL1* which encodes a calcium binding protein and found to be upregulated during bone matrix formation following mechanical loading of bone in rats (Mantila Roosa et al., 2011), *PTH* with its well established roles in regulating calcium homeostasis and bone remodeling (Lombardi et al., 2011), and three long non-coding RNAs with unknown function in bone metabolism (*LINC01476, LINC00301,* and *LINC01699*)*.*

In addition, we also found several other FDR-significant CpG sites, mapping to genes with key bone functions, that were strongly associated with PDB severity based on the site-level analysis. The *GNAS* gene overlaps with a complex locus whose main product is the heterotrimeric G-protein alpha subunit, a key player in the adenylyl cyclase signal pathway. Activating mutations in *GNAS* have been reported to be causative of fibrous dysplasia of bone, a condition characterized by an abnormal differentiation of skeletal progenitor cells with increased osteoclastogenesis, leading to focal osteolytic lesions (Riminucci et al., 2010). We found the methylation level at position chr20:57463767 within the coding part of *GNAS* was increased with disease severity. This finding is in agreement with previously noted hypermethylation of a neighboring CpG site (chr20:57413436) from comparison of PDB cases and controls from the same cohort (Diboun et al., 2021). Increased methylation within the coding part of a gene usually serves to activate its expression and we therefore speculate that *GNAS* is upregulated at the advanced stages of PDB. A similar pattern of CpG hypermethylation within the coding region was observed with *LIF* and *SMO* (Table 2). LIF, the interleukin six class cytokine, is an inhibitor of osteoclast differentiation but has also a stimulatory effect on bone formation (Lorenzo et al., 1990). Moreover, *SMO* which plays an important role in osteoblast differentiation and involved in ciliary cargo transport as part of the osteogenic hedgehog’s proteins signal transduction pathway (Jiang et al., 2013).

We also found hypomethylated FDR-significant CpG sites within upstream promoter regions of key bone related genes including: *OCSTAMP*, *RECQL4* and *DGKZ. OCSTAMP* encodes the osteoclast stimulatory transmembrane protein associated with increased osteoclast nucleation, osteoclast differentiation and osteoclast-mediated bone resorption (Yang et al., 2008). *RECQL4* encodes the RECQ DNA helicase which plays a role in osteoblast formation. Mutations in *RECQL4* have been associated with low bone mass and osteosarcoma (Ng et al., 2015). In addition, *DGKZ* encodes DGKZ enzyme that is thought to modulate the activity of protein kinase C by regulating the levels of diacylglycerols. In bones, DGKZ is thought to have an oncogenic effect by promoting osteosarcoma (Yu et al., 2018) and may play a key role in osteoclast differentiation (Iwazaki et al., 2017). Of note, osteosarcoma develops in some cases of advanced stages of PDB and our data showed decreased methylation in the promotor region of *DGKZ* with increased disease severity. Lastly, we found an FDR-significant positive correlation between promoter located CpG sites and PDB severity with *H19* and *HYAL2*, presumably indicating a decline in the expression of these genes. *H19* encodes a non-coding RNA that is involved in mediating osteogenesis in response to mechanical tension in human bone marrow mesenchymal stem cells (Wu et al., 2018), while *HYAL2* knock out mice develop abnormal craniovertebral bone in addition to hematological anomalies (Jadin et al., 2008).

At the level of regions, CpG islands with the most compelling evidence of association with PDB severity were mapped to *VCL*, *TBX5*, *CASZ1*, *ULBP2*, *NUDT15* and *SQSTM1*. *VCL* encodes a cytoskeletal protein, which mediates the integrin-actin interface to anchor actin filaments to the cell membrane. Downregulation of *VCL* expression is thought to impair cell adhesion and was shown to reduce osteoclastic bone resorption (Fukunaga et al., 2014). In our study, CpG island overlapping the *VCL* gene, showed Bonferroni level of association with disease severity. The most notable amongst all sites in the island was cg00995584 from upstream promoter region (Figure 3). Notably, the chromatin state from this region is predicted to be an active promoter as inferred by ChromHMM (Ernst and Kellis, 2017). A negative association between cg00995584 methylation with disease severity was observed and implies an upregulation of *VCL* with advanced stages of PDB. The other interesting finding from island level analysis was pertaining to an island located within *SQSTM1* gene body. It has long been established that *SQSTM1* plays a central role in the pathology of PDB with the P392L mutation being the most frequently observed amongst familial PDB patients (Ralston and Albagha, 2014). In this study, PDB cases with high disease severity score featured a hypomethylation from *SQSTM1* gene body site cg17987505 (Figure 4). It follows that *SQSTM1* expression could be suppressed in more severe cases of PDB, consistent with a recent report showing that *SQSTM1* deficiency promotes osteoclastogenesis and leads to PDB-like phenotype in mice (Zach et al., 2018). Four more islands, with Bonferroni level of association with PDB severity, were found. These were mapped to: *TBX5* which is associated with osteochondrosis from GWAS studies (Rangkasenee et al., 2013), *CASZ1* which is downregulated during osteoclast differentiation (Toor et al., 2021), *ULBP2* which encodes a major histocompatibility complex protein that is involved in immune system through activation of natural killer cells, and *NUDT15* which is upregulated during osteoclast differentiation (Toor et al., 2021).

Analyzing the global methylation patterns from gene body regions identified two Bonferroni significant associations with PDB severity involving *FYCO1* and *DENND1A*. *FYCO1* acts as the effector of Rab7, a small GTPase, in mediating microtubular transport of vesicles during autophagy. This pathway has been shown to be critical for osteoclastic bone resorption, both for the assembly of osteoclast-bone interface ciliary domains as well as the bone lysis activity that takes place within (Itzstein et al., 2011). Another gene related to the GTPase pathway (*ARHGEF10*) was found to be differentially methylated in PDB cases compared to matched controls in a previous study (Diboun et al., 2021). DENND1 protein is able to partner with clathrin, which in osteoclasts regulates the endocytosis of the master regulator of osteoclastic differentiation and activation, RANKL (Narducci et al., 2010). At the less stringent FDR level, the gene body association analysis uncovered a multitude of genes, of which the most important were *SQSTM1*, *RIN3* and *CSF1*; previously implicated in the pathology of PDB through genetic and functional studies (Chamoux et al., 2009; Albagha et al., 2011; Daroszewska et al., 2011; Albagha et al., 2013; Vallet et al., 2015; Vallet et al., 2021).

Amongst all promoter regions analyzed for associations with PDB severity, the most important were the Bonferroni hits *RPL27*, *LINC00301* and *VPS29*. The *RPL27* gene encodes the ribosomal protein L27. Although there is no literature to support a direct role in bone homeostasis, loss of function mutations in this gene have been reported in patients with Diamond-Blackfan who have an increased risk of developing malignancies including leukaemia and osteosarcoma (Wang et al., 2015). Moreover, *LINC00301* has been recently reported to play a role in tumor progression (Sun et al., 2020). *VPS29* is part of the retromer complex that mediates retrograde transport from endosome to Golgi. VPS29 is thought to play a pivotal role in Wnt signaling pathway by mediating the recycling of WLS receptors back to Golgi (Mehta et al., 2021).

Functional annotations using GO enrichment analysis highlighted two main pathways associated with disease severity; those related to osteoclast proliferation/differentiation and pathways related to immune processes. IPA annotations provided further insights into immune-related pathways. These two pathways were also enriched from the analysis of differentially methylated genes in PDB compared to controls (Diboun et al., 2021) which further highlight their role in the pathogenesis of PDB. Notably, osteoimmunology can have profound effects on bone metabolism (Walsh et al., 2006) and has been linked with PDB (Numan et al., 2015). However, deciphering the imbalances in immune response/pathways under the influence of epigenetic mechanisms in PDB require further investigations.

Our multivariate predictor of PDB severity based on the 2,247 pooled sites from the discovery was highly discriminatory (AUC = 0.8) when tested on the cross-validation set. The Glmnet best subset analysis offered a reasonable compromise between the discriminatory capacity of the model and the number of features (AUC = 0.7, *n* = 33 CpG sites). However, certain limitations of this work must be considered for further research. Firstly, the clinical translation of the devised predictor may be explored further such as including healthy subjects in the analyses, which could increase the specificity of the model by selecting sites exclusively modified in PDB patients. Secondly, although many loci showed high concordance between blood and bone, the analyses were performed on blood and not on bone. Moreover, some of the CpG sites associated with PDB severity were found to regulate gene expression of neighboring genes based on data from blood tissue, however, epigenetic regulation of gene expression is tissue specific. Therefore, to confirm these findings, the analyses could be replicated on specimens from bone tissue of PDB patients. Additionally, while our study is the first to show associations of specific epigenetic DNA methylation patterns with PDB severity, further validation in an independent and possibly larger cohort will be required. Our analysis was based on the illumina HumanMethylation 450K array which covers around 480,000 CpG sites representing around 2% of known genome-wide CpG sites. Therefore, future studies using whole genome bisulphite sequencing provides more comprehensive coverage and could reveal additional differentially methylation loci associated with disease severity. Another limitation of this study is that data on comorbidities such as diabetes were not available, however there is no evidence to indicate that co-morbidities influence severity or development of PDB so we feel it is unlikely that differences in these characteristics could explain the results. Lastly, the identified associations could be a consequence of disease pathogenesis and therefore further prospective studies assessing these changes from disease onset will be required.

## 5 Conclusion

In conclusion, our study identified for the first time a list of DNA methylation sites that were associated with severity of PDB. The list included sites that were within or near genes with important role in bone cell function and metabolism. The study also highlighted novel molecular pathways associated with disease progression such as immune- and viral-related pathways. The identified CpG sites showed a significant correlation between observed and predicted PDB severity scores. This suggests that these sites may be useful as a predictive tool for assessing disease severity of PDB in newly diagnosed patients or patients at risk of developing PDB. Consequently, improved knowledge about the epigenetic factors at play in PDB patients can also support the utilization of epigenetic modifiers for therapeutic benefits in PDB, but further investigations are required.

## Data Availability

The datasets presented in this study can be found in online repositories. The names of the repository/repositories and accession number(s) can be found below: Gene Expression Omnibus, accession number: GSE201322.

## References

[B1] Adachi J. D. Arlen D. Webber C. E. Chettle D. R. Beaumont L. F. Gordon C. L. (1998). Is There Any Association between the Presence of Bone Disease and Cumulative Exposure to Lead? Calcif. Tissue Int. 63 (5), 429–432. 10.1007/s002239900552 9799829

[B2] Albagha O. M. Wani S. E. Visconti M. R. Alonso N. Goodman K. Brandi M. L. (2011). Genome-wide Association Identifies Three New Susceptibility Loci for Paget's Disease of Bone. Nat. Genet. 43 (7), 685–689. 10.1038/ng.845 21623375

[B3] Albagha O. M. E. Visconti M. R. Alonso N. Langston A. L. Cundy T. Dargie R. (2010). Genome-wide Association Study Identifies Variants at CSF1, OPTN and TNFRSF11A as Genetic Risk Factors for Paget's Disease of Bone. Nat. Genet. 42 (6), 520–524. 10.1038/ng.562 20436471 PMC3217192

[B4] Albagha O. M. Visconti M. R. Alonso N. Wani S. Goodman K. Fraser W. D. (2013). Common Susceptibility Alleles andSQSTM1mutations Predict Disease Extent and Severity in a Multinational Study of Patients with Paget's Disease. J. Bone Min. Res. 28 (11), 2338–2346. 10.1002/jbmr.1975 23658060

[B5] Audet M.-C. Jean S. Beaudoin C. Guay-Bélanger S. Dumont J. Brown J. P. (2017). Environmental Factors Associated with Familial or Non-familial Forms of Paget's Disease of Bone. Jt. Bone Spine 84 (6), 719–723. 10.1016/j.jbspin.2016.11.010 27932202

[B6] Barker D. J. P. Gardner M. J. (1974). Distribution of Paget's Disease in England, Wales and Scotland and a Possible Relationship with Vitamin D Deficiency in Childhood. J. Epidemiol. Community Health 28 (4), 226–232. 10.1136/jech.28.4.226 PMC478867 4455340

[B7] Bonder M. J. Luijk R. Luijk R. Zhernakova D. V. Moed M. Deelen P. (2017). Disease Variants Alter Transcription Factor Levels and Methylation of Their Binding Sites. Nat. Genet. 49 (1), 131–138. 10.1038/ng.3721 27918535

[B8] Chamoux E. Couture J. Bisson M. Morissette J. Brown J. P. Roux S. (2009). The P62 P392L Mutation Linked to Paget's Disease Induces Activation of Human Osteoclasts. Mol. Endocrinol. 23 (10), 1668–1680. 10.1210/me.2009-0066 19589897 PMC5224938

[B9] Cronin O. Subedi D. Forsyth L. Goodman K. Lewis S. C. Keerie C. (2020). Characteristics of Early Paget's Disease in SQSTM1 Mutation Carriers: Baseline Analysis of the ZiPP Study Cohort. J. Bone Min. Res. 35 (7), 1246–1252. 10.1002/jbmr.4007 32176830

[B10] Daroszewska A. van 't Hof R. J. Rojas J. A. Layfield R. Landao-Basonga E. Rose L. (2011). A Point Mutation in the Ubiquitin-Associated Domain of SQSMT1 Is Sufficient to Cause a Paget's Disease-like Disorder in Mice. Hum. Mol. Genet. 20 (14), 2734–2744. 10.1093/hmg/ddr172 21515589

[B11] Diboun I. Wani S. Ralston S. H. Albagha O. M. (2021). Epigenetic Analysis of Paget's Disease of Bone Identifies Differentially Methylated Loci that Predict Disease Status. Elife 10, 10. 10.7554/eLife.65715 PMC8184208 33929316

[B12] Ducy P. Amling M. Takeda S. Priemel M. Schilling A. F. Beil F. T. (2000). Leptin Inhibits Bone Formation through a Hypothalamic Relay. Cell. 100 (2), 197–207. 10.1016/s0092-8674(00)81558-5 10660043

[B13] Duncan P. I. Stojdl D. F. Marius R. M. Scheit K. H. Bell J. C. (1998). The Clk2 and Clk3 Dual-Specificity Protein Kinases Regulate the Intranuclear Distribution of SR Proteins and Influence Pre-mRNA Splicing. Exp. Cell. Res. 241 (2), 300–308. 10.1006/excr.1998.4083 9637771

[B14] Ebrahimi P. Luthman H. McGuigan F. E. Akesson K. E. (2021). Epigenome-wide Cross-Tissue Correlation of Human Bone and Blood DNA Methylation - Can Blood Be Used as a Surrogate for Bone? Epigenetics 16 (1), 92–105. 10.1080/15592294.2020.1788325 32692944 PMC7889104

[B15] Ernst J. Kellis M. (2017). Chromatin-state Discovery and Genome Annotation with ChromHMM. Nat. Protoc. 12 (12), 2478–2492. 10.1038/nprot.2017.124 29120462 PMC5945550

[B16] Friedman J. Hastie T. Tibshirani R. (2010). Regularization Paths for Generalized Linear Models via Coordinate Descent. J. Stat. Softw. 33 (1), 1–22. 10.18637/jss.v033.i01 20808728 PMC2929880

[B17] Fujita H. Ochi M. Ono M. Aoyama E. Ogino T. Kondo Y. (2019). Glutathione Accelerates Osteoclast Differentiation and Inflammatory Bone Destruction. Free Radic. Res. 53 (2), 226–236. 10.1080/10715762.2018.1563782 30741054

[B18] Fukunaga T. Zou W. Warren J. T. Teitelbaum S. L. (2014). Vinculin Regulates Osteoclast Function. J. Biol. Chem. 289 (19), 13554–13564. 10.1074/jbc.m114.550731 24675074 PMC4036361

[B19] Gasper T. M. (1979). Paget's Disease in a Treadle Machine Operator. Bmj 1 (6172), 1217–1218. 10.1136/bmj.1.6172.1217-e PMC1599350 445028

[B20] Hocking L. J. Herbert C. A. Nicholls R. K. Williams F. Bennett S. T. Cundy T. (2001). Genomewide Search in Familial Paget Disease of Bone Shows Evidence of Genetic Heterogeneity with Candidate Loci on Chromosomes 2q36, 10p13, and 5q35. Am. J. Hum. Genet. 69 (5), 1055–1061. 10.1086/323798 11555792 PMC1274352

[B21] Holloway W. R. Collier F. M. Aitken C. J. Myers D. E. Hodge J. M. Malakellis M. (2002). Leptin Inhibits Osteoclast Generation. J. Bone Min. Res. 17 (2), 200–209. 10.1359/jbmr.2002.17.2.200 11811550

[B22] Houseman E. A. Accomando W. P. Koestler D. C. Christensen B. C. Marsit C. J. Nelson H. H. (2012). DNA Methylation Arrays as Surrogate Measures of Cell Mixture Distribution. BMC Bioinforma. 13, 86. 10.1186/1471-2105-13-86 PMC3532182 22568884

[B23] Itzstein C. Coxon F. P. Rogers M. J. (2011). The Regulation of Osteoclast Function and Bone Resorption by Small GTPases. Small GTPases 2 (3), 117–130. 10.4161/sgtp.2.3.16453 21776413 PMC3136942

[B24] Iwazaki K. Tanaka T. Hozumi Y. Okada M. Tsuchiya R. Iseki K. (2017). DGKζ Downregulation Enhances Osteoclast Differentiation and Bone Resorption Activity under Inflammatory Conditions. J. Cell. Physiol. 232 (3), 617–624. 10.1002/jcp.25461 27312515

[B25] Jadin L. Wu X. Ding H. Frost G. I. Onclinx C. Triggs‐Raine B. (2008). Skeletal and Hematological Anomalies in HYAL2‐deficient Mice: a Second Type of Mucopolysaccharidosis IX? FASEB J. 22 (12), 4316–4326. 10.1096/fj.08-111997 18772348

[B26] Jiang Q. Du J. Yin X. Shan Z. Ma Y. Ma P. (2013). Shh Signaling, Negatively Regulated by BMP Signaling, Inhibits the Osteo/dentinogenic Differentiation Potentials of Mesenchymal Stem Cells from Apical Papilla. Mol. Cell. Biochem. 383 (1-2), 85–93. 10.1007/s11010-013-1757-9 23867990

[B27] Karsenty G. (2006). Convergence between Bone and Energy Homeostases: Leptin Regulation of Bone Mass. Cell. Metab. 4 (5), 341–348. 10.1016/j.cmet.2006.10.008 17084709

[B28] Laurin N. Brown J. P. Lemainque A. Duchesne A. Huot D. Lacourcière Y. (2001). Paget Disease of Bone: Mapping of Two Loci at 5q35-Qter and 5q31. Am. J. Hum. Genet. 69 (3), 528–543. 10.1086/322975 11473345 PMC1235483

[B29] Lombardi G. Di Somma C. Rubino M. Faggiano A. Vuolo L. Guerra E. (2011). The Roles of Parathyroid Hormone in Bone Remodeling: Prospects for Novel Therapeutics. J. Endocrinol. Investig. 34 (7 Suppl. l), 18–22. 21985975

[B30] Lorenzo J. A. Sousa S. L. Leahy C. L. (1990). Leukemia Inhibitory Factor (LIF) Inhibits Basal Bone Resorption in Fetal Rat Long Bone Cultures. Cytokine 2 (4), 266–271. 10.1016/1043-4666(90)90027-q 2129503

[B31] Mantila Roosa S. M. Liu Y. Turner C. H. (2011). Gene Expression Patterns in Bone Following Mechanical Loading. J. Bone Min. Res. 26 (1), 100–112. 10.1002/jbmr.193 PMC3179310 20658561

[B32] Martin T. C. Yet I. Tsai P.-C. Bell J. T. (2015). coMET: Visualisation of Regional Epigenome-wide Association Scan Results and DNA Co-methylation Patterns. BMC Bioinforma. 16, 131. 10.1186/s12859-015-0568-2 PMC4422463 25928765

[B33] Mehta S. Hingole S. Chaudhary V. (2021). The Emerging Mechanisms of Wnt Secretion and Signaling in Development. Front. Cell. Dev. Biol. 9, 714746. 10.3389/fcell.2021.714746 34485301 PMC8415634

[B34] Moore L. D. Le T. Fan G. (2013). DNA Methylation and its Basic Function. Neuropsychopharmacol 38 (1), 23–38. 10.1038/npp.2012.112 PMC3521964 22781841

[B35] Müller F. Scherer M. Assenov Y. Lutsik P. Walter J. Lengauer T. (2019). RnBeads 2.0: Comprehensive Analysis of DNA Methylation Data. Genome Biol. 20 (1), 55. 10.1186/s13059-019-1664-9 30871603 PMC6419383

[B36] Narducci P. Bortul R. Bareggi R. Nicolin V. (2010). Clathrin-dependent Endocytosis of Membrane-Bound RANKL in Differentiated Osteoclasts. Eur. J. Histochem 54 (1), e6. 10.4081/ejh.2010.e6 20353913 PMC3167292

[B37] Ng A. J. M. Walia M. K. Smeets M. F. Mutsaers A. J. Sims N. A. Purton L. E. (2015). The DNA Helicase Recql4 Is Required for Normal Osteoblast Expansion and Osteosarcoma Formation. PLoS Genet. 11 (4), e1005160. 10.1371/journal.pgen.1005160 25859855 PMC4393104

[B38] Numan M. S. Amiable N. Brown J. P. Michou L. (2015). Paget's Disease of Bone: an Osteoimmunological Disorder? Drug Des. Devel Ther. 9, 4695–4707. 10.2147/DDDT.S88845 PMC4544727 26316708

[B39] Obaid R. Wani S. E. Azfer A. Hurd T. Jones R. Cohen P. (2015). Optineurin Negatively Regulates Osteoclast Differentiation by Modulating NF-Κb and Interferon Signaling: Implications for Paget's Disease. Cell. Rep. 13 (6), 1096–1102. 10.1016/j.celrep.2015.09.071 26527009 PMC4646838

[B40] Pidsley R.C. C. Y. W. Y Wong M. Lunnon K. Mill J. Schalkwyk L. C. (2013). A Data-Driven Approach to Preprocessing Illumina 450K Methylation Array Data. BMC Genomics 14, 293. 10.1186/1471-2164-14-293 23631413 PMC3769145

[B41] Piekarz R. L. Bates S. E. (2009). Epigenetic Modifiers: Basic Understanding and Clinical Development. Clin. Cancer Res. 15 (12), 3918–3926. 10.1158/1078-0432.ccr-08-2788 19509169 PMC6946182

[B42] Ralston S. H. Albagha O. M. E. (2014). Genetics of Paget's Disease of Bone. Curr. Osteoporos. Rep. 12 (3), 263–271. 10.1007/s11914-014-0219-y 24988994

[B43] Ralston S. H. (2013). Paget's Disease of Bone. N. Engl. J. Med. 368 (7), 644–650. 10.1056/nejmcp1204713 23406029

[B44] Ralston S. H. (2008). Pathogenesis of Paget's Disease of Bone. Bone 43 (5), 819–825. 10.1016/j.bone.2008.06.015 18672105

[B45] Rangkasenee N. Murani E. Brunner R. M. Schellander K. Cinar M. U. Luther H. (2013). Genome-Wide Association Identifies TBX5 as Candidate Gene for Osteochondrosis Providing a Functional Link to Cartilage Perfusion as Initial Factor. Front. Genet. 4, 78. 10.3389/fgene.2013.00078 23675383 PMC3650520

[B46] Riminucci M. Gehron Robey P. Saggio I. Bianco P. (2010). Skeletal Progenitors and the GNAS Gene: Fibrous Dysplasia of Bone Read through Stem Cells. J. Mol. Endocrinol. 45 (6), 355–364. 10.1677/jme-10-0097 20841428 PMC3384548

[B47] Robertson K. D. (2005). DNA Methylation and Human Disease. Nat. Rev. Genet. 6 (8), 597–610. 10.1038/nrg1655 16136652

[B48] Singer F. R. (2015). Paget's Disease of Bone-Genetic and Environmental Factors. Nat. Rev. Endocrinol. 11 (11), 662–671. 10.1038/nrendo.2015.138 26284446

[B49] Siris E. S. (1994). Epidemiological Aspects of Paget's Disease: Family History and Relationship to Other Medical Conditions. Seminars Arthritis Rheumatism 23 (4), 222–225. 10.1016/0049-0172(94)90037-x 8009230

[B50] Smyth G. K. (2004). Linear Models and Empirical Bayes Methods for Assessing Differential Expression in Microarray Experiments. Stat. Appl. Genet. Mol. Biol. 3, Article3. 10.2202/1544-6115.1027 16646809

[B51] Sun C.-C. Zhu W. Li S.-J. Hu W. Zhang J. Zhuo Y. (2020). FOXC1-mediated LINC00301 Facilitates Tumor Progression and Triggers an Immune-Suppressing Microenvironment in Non-small Cell Lung Cancer by Regulating the HIF1α Pathway. Genome Med. 12 (1), 77. 10.1186/s13073-020-00773-y 32878637 PMC7466809

[B52] Tan A. Goodman K. Walker A. Hudson J. MacLennan G. S. Selby P. L. (2017). Long-Term Randomized Trial of Intensive versus Symptomatic Management in Paget's Disease of Bone: The PRISM-EZ Study. J. Bone Min. Res. 32 (6), 1165–1173. 10.1002/jbmr.3066 28176386

[B53] Toor S. M. Wani S. Albagha O. M. E. (2021). Comprehensive Transcriptomic Profiling of Murine Osteoclast Differentiation Reveals Novel Differentially Expressed Genes and LncRNAs. Front. Genet. 12, 781272. 10.3389/fgene.2021.781272 34868271 PMC8634834

[B54] Vallet M. Soares D. C. Wani S. Sophocleous A. Warner J. Salter D. M. (2015). Targeted Sequencing of the Paget's Disease Associated 14q32 Locus Identifies Several Missense Coding Variants in RIN3 that Predispose to Paget's Disease of Bone. Hum. Mol. Genet. 24 (11), 3286–3295. 10.1093/hmg/ddv068 25701875 PMC4424954

[B55] Vallet M. Sophocleous A. Törnqvist A. E. Azfer A. Hof R. v. t. Albagha O. M. (2021). Targeted Inactivation of Rin3 Increases Trabecular Bone Mass by Reducing Bone Resorption and Favouring Bone Formation. Calcif. Tissue Int. 109 (1), 92–102. 10.1007/s00223-021-00827-2 33725152 PMC8225545

[B56] Visconti M. R. Langston A. L. Alonso N. Goodman K. Selby P. L. Fraser W. D. (2010). Mutations ofSQSTM1are Associated with Severity and Clinical Outcome in Paget Disease of Bone. J. Bone Min. Res. 25 (11), 2368–2373. 10.1002/jbmr.132 20499339

[B57] Visconti M. R. Usategui-Martín R. Ralston S. H. (2017). Antibody Response to Paramyxoviruses in Paget's Disease of Bone. Calcif. Tissue Int. 101 (2), 141–147. 10.1007/s00223-017-0265-4 28361207 PMC5498588

[B58] Walsh M. C. Kim N. Kadono Y. Rho J. Lee S. Y. Lorenzo J. (2006). Osteoimmunology: Interplay between the Immune System and Bone Metabolism. Annu. Rev. Immunol. 24, 33–63. 10.1146/annurev.immunol.24.021605.090646 16551243

[B59] Wang R. Yoshida K. Toki T. Sawada T. Uechi T. Okuno Y. (2015). Loss of Function Mutations inRPL27andRPS27identified by Whole-Exome Sequencing in Diamond-Blackfan Anaemia. Br. J. Haematol. 168 (6), 854–864. 10.1111/bjh.13229 25424902

[B60] Wani S. Daroszewska A. Salter D. M. van 't Hof R. J. Ralston S. H. Albagha O. M. E. (2022). The Paget's Disease of Bone Risk Gene PML is a Negative Regulator of Osteoclast Differentiation and Bone Resorption. Dis. Model. Mech. 15 15 (4). 10.1242/dmm.049318 PMC9066519 35229101

[B61] Wong S.-W. Huang B.-W. Hu X. Kim E. H. Kolb J. P. Padilla R. J. (2021). Correction: Global Deletion of Optineurin Results in Altered Type I IFN Signaling and Abnormal Bone Remodeling in a Model of Paget's Disease. Cell. Death Differ. 28 (2), 825–826. 10.1038/s41418-020-0586-0 32678306 PMC7862615

[B62] Wu J. Zhao J. Sun L. Pan Y. Wang H. Zhang W.-B. (2018). Long Non-coding RNA H19 Mediates Mechanical Tension-Induced Osteogenesis of Bone Marrow Mesenchymal Stem Cells via FAK by Sponging miR-138. Bone 108, 62–70. 10.1016/j.bone.2017.12.013 29253550

[B63] Yang M. Birnbaum M. J. MacKay C. A. Mason-Savas A. Thompson B. Odgren P. R. (2008). Osteoclast Stimulatory Transmembrane Protein (OC-STAMP), a Novel Protein Induced by RANKL that Promotes Osteoclast Differentiation. J. Cell. Physiol. 215 (2), 497–505. 10.1002/jcp.21331 18064667 PMC2762860

[B64] Yu W. Tang L. Lin F. Yao Y. Shen Z. (2018). DGKZ Acts as a Potential Oncogene in Osteosarcoma Proliferation through its Possible Interaction with ERK1/2 and MYC Pathway. Front. Oncol. 8, 655. 10.3389/fonc.2018.00655 30662872 PMC6328465

[B65] Zach F. Polzer F. Mueller A. Gessner A. (2018). p62/sequestosome 1 Deficiency Accelerates Osteoclastogenesis *In Vitro* and Leads to Paget's Disease-like Bone Phenotypes in Mice. J. Biol. Chem. 293 (24), 9530–9541. 10.1074/jbc.ra118.002449 29555685 PMC6005453

[B66] Zhou Y. Yang L. Wang H. Chen X. Jiang W. Wang Z. (2020). Alterations in DNA Methylation Profiles in Cancellous Bone of Postmenopausal Women with Osteoporosis. FEBS Open Bio 10 (8), 1516–1531. 10.1002/2211-5463.12907 PMC7396431 32496000

